# Social inequalities in the provision of obstetric services in Norway 1967–2009: a population-based cohort study

**DOI:** 10.1093/eurpub/ckaa007

**Published:** 2020-02-07

**Authors:** Helene Sofie Eriksen, Susanne Høy, Lorentz M Irgens, Svein Rasmussen, Kjell Haug

**Affiliations:** c1 Department of Internal Medicine, Ringerike Hospital, Vestre Viken Hospital Trust, Hønefoss, Norway; c2 Department of Surgery, Lillehammer Hospital, Innlandet Hospital Trust, Lillehammer, Norway; c3 Department of Global Public Health and Primary Care, University of Bergen, Bergen, Norway; c4 Medical Birth Registry of Norway, Norwegian Institute of Public Health, Bergen, Norway; c5 Department of Clinical Science, University of Bergen, Bergen, Norway; c6 Department of Obstetrics and Gynecology, Haukeland University Hospital, Bergen, Norway

## Abstract

**Background:**

Socioeconomic (SE) inequalities have been observed in a number of adverse outcomes of pregnancy and many of the risk factors for such outcomes are associated with a low SE level. However, SE inequalities persist even after adjustment for these risk factors. Less well-off women are more vulnerable, but may also get less adequate health services. The objective of the present study was to assess possible associations between SE conditions in terms of maternal education as well as ethnic background and obstetric care.

**Methods:**

A population-based national cohort study from the Medical Birth Registry of Norway. The study population comprised 2 305 780 births from the observation period 1967–2009. Multilevel analysis was used because of the hierarchical structure of the data. Outcome variables included induction of labour, epidural analgesia, caesarean section, neonatal intensive care and perinatal death.

**Results:**

While medical interventions in the 1970s were employed less frequently in women of short education and non-western immigrants, this difference was eliminated or even reversed towards the end of the observation period. However, an excess perinatal mortality in both the short-educated [adjusted relative risk (aRR) = 2.49] and the non-western immigrant groups (aRR = 1.75) remained and may indicate increasing health problems in these groups.

**Conclusion:**

Even though our study suggests a fair and favourable development during the last decades in the distribution across SE groups of obstetric health services, the results suggest that the needs for obstetric care have increased in vulnerable groups, requiring a closer follow-up.

## Introduction

Over a long period of time, numerous studies in developed countries have reported substantial socioeconomic (SE) inequalities in morbidity and mortality, irrespective of how SE inequalities have been measured.^1–3^ In the interpretation of the results, emphasis has been put on inequalities in the exposure to the putative risk factors. The conclusion has been drawn that SE factors, such as educational level, may represent a proxy variable for material, psychosocial and behavioural risk factors.^4–6^

However, registered morbidity and mortality are not only dependent on risk factors, but also on the health services provided.^7^^,^^8^ From an ethical point of view, substantial SE differences in the occurrence of diseases are unacceptable, but SE differences in the provision of health services in favour of well-off groups are intolerable. Medico-ethical rules have recommended that health services should be provided according to need rather than social background. Still, recent legislation in Norway, one of the world’s most egalitarian countries,^9^ has found it necessary to emphasize equal provision of health services irrespective of social background.^10^

Also in adverse outcomes of pregnancy, substantial SE inequalities have been observed in a number of countries.^11–16^ Even with a perinatal mortality in Norway in 2011 of 4.8 per 1000 births,^17^ substantial differences have been reported between social groups.^18^ Many of the risk factors for adverse outcomes of pregnancy are associated with a low SE level.^5^^,^^16^^,^^19^^,^^20^ However, SE inequalities persist even after adjustment for these risk factors, suggesting the influence of other factors not accounted for.

Only few studies have addressed the question to which extent inequality in the provision of health services can account for inequality in health.^21–23^ Less well-off women may get less adequate health services.

Another vulnerable category of mothers is the increasing group of immigrants to western countries.^24^^,^^25^ Irrespective of their length of education or SE status in their country of origin, they may experience poor living conditions with a limited network and language difficulties.

Use of interventions is related to pathological conditions with different occurrence in different SE groups. By analysing subgroups with the same occurrence of pathology, valid estimates of possible effects of short education or status as immigrant on use of obstetrical interventions may be obtained. One such subgroup is preterm birth in which degree of pathology seems to be more independent of SE conditions than in term birth.^26^^,^^27^ Accordingly, need of intervention in preterm birth would be expected to be more independent of SE level.

A generation ago, long education among women was less common than today. As a group, women with short education were more heterogeneous. Today, women with short education represent a smaller and more homogeneous group with a higher burden of risk factors. Therefore, we might expect more health problems and more interventions among these women.

The objective of the present study was to assess possible associations between SE conditions as well as ethnic background and obstetric care. Based on data from the Medical Birth Registry of Norway, we wanted to assess to which extent provision of various types of obstetric intervention is dependent on the parent’s SE level measured in terms of length of education. We also wanted to assess whether immigrants are provided with less adequate obstetric care.

A possible secular trend in the use of obstetric interventions across educational groups might also reflect attitudes among professionals that change over a period of time. Thus, we studied time trends in the associations of level of education and ethnic background with obstetric interventions and perinatal mortality throughout the observation period 1967–2009.

## Methods

Based on compulsory notification, the Medical Birth Registry of Norway has, since 1967, registered all pregnancies in the country. Items of personal data include the national identification number as well as medical data on maternal health before and during the pregnancy, on the delivery and on the newborn. All data are notified by the attending midwife and doctor.^28^ The national identification number provides linkage with the Statistics Norway for data on parental education and country of birth.

During the observation period 1967–2009 altogether 2 518 758 births were notified. After exclusion of multiple births and birthweight under 500 g or gestational age under 22 weeks, the study population comprised 2 305 780 births. After further exclusion of births with lacking data on both parental education and country of birth, and births with gestational age 43+ weeks, the main analyses included 2 234 568 births.

Exposure variables included education defined as the highest level of education for the mother or the father obtained by 2009 and categorized as (i) university or college (more than 14 years), (ii) intermediate (11–14 years) and (iii) none or no more than compulsory education (0–10 years). All births to mothers who were born in Norway, Western Europe, the USA, Australia and New Zealand were categorized according to the highest parental level of education. As we also wanted to assess whether non-western born mothers were provided with an adequate obstetric care, all these women were included in a group 4 irrespective of their educational attainment. Since this group was small until the 1980s, we do not report complete data for the first period 1967–80.

Outcome variables included induction of labour, epidural analgesia, caesarean section (CS), transfer of the newborn to a neonatal intensive care unit (NICU) and perinatal death.

### Statistical analyses

For statistical analysis, we used SPSS (version 20) and the MlWin programme (version 2.30). Multilevel analysis was used due to the hierarchical structure (the first level was the pregnancy and the second level was the mother). The highest level of parental education was used as reference.

The analyses were stratified according to gestational age categorized as preterm (22–36 weeks) and term (37–42 weeks) and adjusted for maternal age categorized as <20, 20–24, 25–29, 30–34, 35–39 and 40+ years and for birth order categorized as 1, 2, 3, 4 and 5+. Further adjustment was made for size of the maternity unit categorized as 1–499, 500–1499, 1500–2999 and 3000+ births per year since they are related to exposure and outcome.

Particularly in the first part of the observation period, a considerable proportion of the births took place in rural smaller hospitals with limited possibilities to provide some of the interventions studied. An association between rural domicile and short education would confound the results. Thus, the relative risks (RRs) were also adjusted for annual number of births in the hospital where the delivery took place.

## Results

The proportion of mothers with the highest level of parental education increased from 29.7% in 1967–80 to 53.7% in 2001–09 while the two other groups decreased correspondingly (table 1). The proportion of mothers born in Western world decreased continuously from 99.2% in 1967–80 to 85.0% in 2001–09.

**Table 1:** Births in Norway 1967–2009 by demographic characteristics and year of birth

	Year of birth/number of births (*n*) and percent (%)				
	1967–80, *n* (%)	1981–90, *n* (%)	1991–2000, *n* (%)	2001–09, *n* (%)	Total (%)
Highest parental level of education					
Education <11 years	106 111 (13.6)	47 182 (9.8)	44 150 (8.2)	47 325 (9.4)	10.6
Education 11–14 years	441 659 (56.4)	232 168 (48.1)	232 945 (43.5)	173 897 (34.4)	46.9
Education ≥15 years	232 497 (29.7)	201 553 (41.7)	255 475 (47.7)	271 399 (53.7)	41.7
Education not recorded	2146 (0.3)	2114 (0.4)	2713 (0.5)	12 446 (2.5)	0.8
Gestational age					
Preterm (22–36 weeks)	41 653 (5.3)	25 696 (5.3)	29 895 (5.6)	27 849 (5.5)	5.4
Term (37–42 weeks)	710 291 (90.8)	439 009 (90.9)	491 464 (91.8)	475 748 (94.2)	91.8
Post term (43+ weeks)	30 469 (3.9)	18 312 (3.8)	13 924 (2.6)	1470 (0.3)	2.8
Maternal age					
<20	74 718 (9.5)	25 998 (5.4)	16 176 (3.0)	11 825 (2.3	5.6
20–24	283 378 (36.2)	134 173 (27.8)	104 240 (19.5)	73 964 (14.6)	25.8
25–29	249 591 (31.9)	180 213 (37.3)	198 748 (37.1)	162 378 (32.1)	34.3
30–34	117 779 (15.1)	104 542 (21.6)	151 221 (28.3)	169 204 (33.5)	23.5
35–39	44 496 (5.7)	33 058 (6.8)	56 027 (10.5)	74 881 (14.8)	9.0
40+	12 451 (1.6)	5033 (1.0)	8871 (1.7)	12 774 (2.5)	1.7
Not recorded	0 (0.0)	0 (0.0)	0 (0.0)	41 (0.0)	0.0
Birth number					
1	319 742 (40.9)	208 169 (43.1)	218 791 (40.9)	208 832 (41.3)	41.4
2	261 903 (33.5)	169 872 (35.2)	190 439 (35.6)	180 191 (35.7)	34.8
3	125 079 (16.0)	76 962 (15.9)	91 257 (17.0)	81 616 (16.2)	16.3
4	47 348 (6.1)	20 079 (4.2)	24 840 (4.6)	23 375 (4.6)	5.0
5+	28 341 (3.6)	7935 (1.6)	9956 (1.9)	11 053 (2.2)	2.5
Mother’s country of birth					
Western world	775 792 (99.2)	466 496 (96.6)	495 201 (92.5)	429 179 (85.0)	94.0
Non-western world	6621 (0.8)	16 521 (3.4)	40 082 (7.5)	75 888 (15.0)	6.0
Maternity unit (births per year)					
1–499	179 201 (22.9)	75 786 (15.7)	58 495 (10.9)	51 538 (10.2)	15.8
500–1499	307 356 (39.3)	168 031 (34.8)	130 753 (24.4)	114 344 (22.6)	31.2
1500–2999	215 349 (27.5)	132 609 (27.5)	167 808 (31.3)	134 188 (26.6)	28.2
3000+	73 489 (9.4)	104 450 (21.6)	174 138 (32.5)	200 584 (39.7)	24.0
Outside institution	7018 (0.9)	2141 (0.4)	4089 (0.8)	4413 (0.9)	0.8
Total	782 413 (100)	483 017 (100)	535 283 (100)	505 067 (100)	100

### Preterm births

In preterm births, during the first part of the observation period 1967–80, women with short education (<11 years) had less frequently induction of labour (RR 0.88), CS (RR 0.91) and epidural analgesia (RR 0.79) and had their newborns less frequently transferred to an NICU (RR 0.81) (table 2). Their offspring also had an excess perinatal mortality (RR 1.13) compared with those with long education (15+ years) (table 4).

**Table 2:** Obstetric services provided in preterm birth (22–36 weeks) according to parental educational level and mothers’ country of origin by year of birth, Norway 1967–2009

	Year of birth/number (*n*), per 1000 births and aRR and 95% confidence interval (CI)											
	1967–80			1981–90			1991–2000			2001–09		
	*n*	Per 1000	aRR (95% CI)	*n*	Per 1000	aRR (95% CI)	*n*	Per 1000	aRR (95% CI)	*n*	Per 1000	aRR (95% CI)
Induction of labour												
Education <11 years	892	118.2	0.88 (0.81–0.97)	653	209.6	1.02 (0.94–1.12)	864	328.6	1.04 (0.96–1.12)	908	422.1	1.08 (1.00–1.16)
Education 11–14 years	2852	121.4	0.92 (0.87–0.99)	2626	214.1	0.99 (0.94–1.06)	4174	331.1	1.03 (0.98–1.07)	3894	427.0	1.05 (1.00–1.09)
Education ≥15 years	1457	145.5	Ref	2054	231.0	Ref	3915	337.8	Ref	5076	431.0	Ref
Non-western				275	205.8	0.86 (0.76–0.98)	932	308.8	0.92 (0.86–0.99)	1903	409.2	0.97 (0.91–1.02)
Caesarean section												
Education <11 years	628	83.2	0.91 (0.83–0.99)	794	254.9	0.94 (0.87–1.02)	838	318.8	1.03 (0.96–1.12)	787	365.9	1.08 (1.00–1.17)
Education 11–14 years	2155	91.7	1.05 (0.97–1.13)	3494	284.9	1.00 (0.95–1.05)	4261	338.0	1.07 (1.03–1.12)	3481	381.7	1.07 (1.02–1.12)
Education ≥15 years	1123	112.1	Ref	2762	310.7	Ref	3881	334.8	Ref	4496	381.8	Ref
Non-western				350	262.0	0.83 (0.74–0.93)	919	304.5	0.92 (0.85–0.99)	1674	359.9	0.98 (0.93–1.04)
Epidural analgesia												
Education <11 years	68	9.0	0.79 (0.60–1.05)	443	142.2	0.99 (0.88–1.10)	433	164.7	0.99 (0.89–1.10)	496	230.6	1.05 (0.95–1.17)
Education 11–14 years	267	11.4	0.81 (0.67–0.97)	1675	136.6	0.92 (0.85–0.98)	2054	162.9	0.96 (0.90–1.02)	1992	218.4	1.06 (0.99–1.12)
Education ≥15 years	212	21.2	Ref	1465	164.8	Ref	2134	184.1	Ref	2477	210.3	Ref
Non-western				145	108.5	0.63 (0.53–0.74)	437	144.8	0.79 (0.71–0.88)	878	188.8	0.87 (0.80–0.94)
Transfer intensive care												
Education <11 years	590	78.2	0.81 (0.74–0.90)	352	113.0	0.94 (0.83–1.05)	469	178.4	0.90 (0.83–0.99)	1194	555.1	1.05 (1.00–1.10)
Education 11–14 years	2104	89.5	0.84 (0.79–0.91)	1480	120.7	0.98 (0.90–1.05)	2458	195.0	0.99 (0.94–1.05)	5101	559.4	1.06 (1.03–1.09)
Education ≥15 years	1280	127.8	Ref	1140	128.2	Ref	2468	212.9	Ref	6318	536.5	Ref
Non-western				96	71.9	0.51 (0.42–0.61)	576	190.9	0.89 (0.82–0.96)	2340	503.1	0.94 (0.91–0.97)

**Table 4:** Perinatal mortality in preterm and term births according to parental educational level and mothers’ country of origin by year of birth, Norway 1967–2009

	Year of birth/number (*n*), per 1000 births and aRR with 95% confidence interval (CI)													
	1967–80			1981–90			1991–2000			2001–09			Total	
	*n*	Per 1000	aRR (CI)	*n*	Per 1000	aRR (CI)	*n*	Per 1000	aRR (CI)	*n*	Per 1000	RR (CI)	*n*	Per 1000
Preterm (22–36 weeks)														
Education <11 years	1525	202.0	1.13 (1.05–1.21)	458	147.0	1.72 (1.52–1.94)	221	84.1	1.43 (1.22–1.67)	146	67.9	1.69 (1.38–2.06)	2350	152.2
Education 11–14 years	4154	176.8	1.02 (0.96–1.08)	1228	100.1	1.18 (1.07–1.29)	756	60.0	1.05 (0.95–1.17)	389	42.7	1.05 (0.92–1.21)	6527	113.5
Education ≥15 years	1745	174.3	Ref	761	85.6	Ref	661	57.0	Ref	497	42.2	Ref	3664	86.7
Non-western	64	132.2	0.73 (0.57–0.94)	114	85.3	0.95 (0.78–1.16)	203	67.3	1.12 (0.95–1.31)	251	54.0	1.23 (1.06–1.44)	632	66.6
Term (37–42 weeks)														
Education <11 years	869	9.4	1.92 (1.74–2.11)	220	5.8	2.31 (1.95–2.73)	127	4.1	1.84 (1.50–2.24)	64	2.6	1.85 (1.40–2.45)	1280	6.9
Education 11–14 years	2696	6.8	1.46 (1.36–1.58)	693	3.4	1.33 (1.18–1.50)	531	2.7	1.18 (1.04–1.34)	323	2.3	1.53 (1.31–1.78)	4243	4.5
Education ≥15 years	1033	4.9	Ref	468	2.6	Ref	539	2.4	Ref	399	1.7	Ref	2439	2.9
Non-western	36	6.1	1.24 (0.89–1.73)	59	4.0	1.52 (1.16–2.00)	136	3.8	1.61 (1.33–1.95)	220	3.1	1.96 (1.65–2.32)	451	3.5
Preterm and term														
Education <11 years	2394	23.5	1.84 (1.74–1.95)	678	16.2	2.60 (2.35–2.87)	348	10.2	2.10 (1.85–2.37)	210	7.9	2.49 (2.12–2.93)	3630	17.8
Education 11–14 years	6850	16.0	1.33 (1.27–1.39)	1921	8.6	1.40 (1.30–1.51)	1287	6.0	1.25 (1.16–1.36)	712	4.7	1.47 (1.33–1.63)	10 770	10.6
Education ≥15 years	2778	12.4	Ref	1229	6.4	Ref	1200	5.1	Ref	896	3.6	Ref	6103	6.8
Non-western	100	15.1	1.15 (0.94–1.41)	173	10.6	1.54 (1.31–1.82)	339	8.5	1.65 (1.46–1.87)	471	6.3	1.75 (1.56–1.96)	1083	7.9

During the last part 2001–09, women with short parental education had more frequently induction of labour (RR 1.08), CS (RR 1.08) and epidural analgesia (1.05) and had their newborns more frequently transferred to an NICU (RR 1.05) (table 2). However, the excess perinatal mortality was even higher than in the first period (RR 1.69) (table 4).

### Term births

In term births during the first period, women with short parental education had their newborn transferred to an NICU, induction of labour and CS more close to the reference group, while epidural analgesia was less frequent (RR 0.69) (table 3). However, during the last period, provision of a number of services increased: transfer to NICU (RR 1.45), induction of labour (RR 1.04), CS (RR 1.36) and epidural analgesia (RR 1.19).

**Table 3:** Obstetric services provided in term birth (37–42 weeks) according to parental educational level and mothers’ country of origin by year of birth, Norway 1967–2009

	Year of birth/number (*n*), per 1000 births and aRR with 95% confidence interval (CI)											
	1967–80			1981–90			1991–2000			2001–09		
	*n*	Per 1000	aRR (95% CI)	*n*	Per 1000	aRR (95% CI)	*n*	Per 1000	aRR (95% CI)	*n*	Per 1000	aRR (95% CI)
Induction of labour												
Education <11 years	11 152	120.6	0.90 (0.88–0.92)	5424	144.2	1.06 (1.03–1.09)	4966	160.7	1.16 (1.12–1.19)	5021	205.3	1.04 (1.02–1.05)
Education 11–14 years	49 680	124.7	0.93 (0.92–0.94)	29 673	144.5	1.03 (1.01–1.04)	30 946	154.9	1.08 (1.06–1.10)	29 181	207.2	1.02 (1.02–1.03)
Education ≥15 years	30 460	143.7	Ref	27 132	150.6	Ref	34 702	155.2	Ref	47 084	198.9	Ref
Non-western	739	125.0	0.87 (0.80–0.93)	2129	145.4	0.97 (0.93–1.02)	5582	154.1	1.05 (1.02–1.08)	13 437	189.5	1.00 (1.00–1.01)
Caesarean section												
Education <11 years	2889	31.2	1.04 (0.99–1.09)	3626	96.4	1.24 (1.19–1.29)	3383	109.5	1.32 (1.27–1.37)	3296	134.8	1.36 (1.31–1.41)
Education 11–14 years	12 855	32.3	1.01 (0.98–1.05)	18 913	92.1	1.09 (1.07–1.12)	20 574	103.0	1.15 (1.13–1.17)	19 888	141.2	1.24 (1.22–1.26)
Education ≥15 years	8326	39.3	Ref	17 272	95.9	Ref	22 759	101.8	Ref	31 386	132.6	Ref
Non-western				2031	138.7	1.59 (1.51–1.67)	4762	131.5	1.47 (1.42–1.52)	11 189	157.8	1.38 (1.35–1.42)
Epidural analgesia												
Education <11 years	465	5.0	0.69 (0.62–0.76)	3473	92.3	1.04 (1.00–1.08)	4126	133.6	1.10 (1.06–1.13)	7674	313.8	1.19 (1.16–1.22)
Education 11–14 years	2392	6.0	0.64 (0.60–0.67)	16 465	80.2	0.91 (0.89–0.93)	24 848	124.4	0.99 (0.98–1.01)	37 329	265.1	1.08 (1.07–1.09)
Education ≥15 years	3128	14.8	Ref	19 002	105.5	Ref	31 883	142.6	Ref	62 617	264.6	Ref
Non-western				1612	110.1	0.95 (0.90–1.00)	5043	139.2	0.97 (0.94–1.00)	17 787	250.9	0.95 (0.94–0.97)
Transfer intensive care												
Education <11 years	703	7.6	0.93 (0.85–1.02)	371	9.9	1.27 (0.13–1.43)	616	19.9	1.11 (1.02–1.21)	1633	66.8	1.45 (1.37–1.53)
Education 11–14 years	3360	8.4	0.95 (0.95–1.01)	1868	9.1	1.13 (1.06–1.22)	3627	18.2	1.02 (0.97–1.07)	8246	58.6	1.25 (1.22–1.29)
Education ≥15 years	2253	10.6	Ref	1570	8.7	Ref	4416	19.7	Ref	12 137	51.3	Ref
Non-western				125	8.5	0.97 (0.81–1.16)	910	25.1	1.30 (1.21–1.41)	3945	55.6	1.11 (1.07–1.16)

The excess perinatal mortality for term births ranged between RR 1.92 and 1.85 throughout the whole observation period 1967–2009 (table 4). In the same period the excess ‘birthweight below 2500 g’ increased from RR 2.19 to RR 2.80 (data not shown).

### Non-western preterm group

In the non-western preterm group, transfer to NICU increased during the study period 1981–2009 (RR from 0.51 to 0.94), and so did induction of labour (RR from 0.86 to 0.97), CS (RR from 0.83 to 0.98) and epidural analgesia (RR from 0.63 to 0.87) (table 2). Perinatal mortality increased during the whole study period 1967–2009 (RR from 0.73 to 1.23) (table 4).

### Non-western term group

In the non-western term group there was an increase in transfer to NICU (RR from 0.97 to 1.11), while the RR of induction of labour and epidural analgesia were close to the reference group while CS decreased (RR from 1.59 to 1.38) throughout the observation period 1981-2009 (table 3). The RR of perinatal mortality increased from 1.24 to 1.96 during the period 1967–2009 (table 4). In the same period the excess ‘birthweight below 2500 g’ ranged between RR 2.34 and RR 2.41 (data not shown).

## Discussion

Our results indicate that in the 1970s, women with short education were provided with less obstetric services than women with long education. During the observation period, the utilization of obstetric services in the short-educated group increased both in preterm as well as term births; in preterm births to the level observed in the longer-educated group, and in term births beyond this level. In non-western women, similar trends were observed.

In an equality of health care perspective, this result is gratifying as the distribution of obstetric services moved in a beneficial direction with more services provided to less privileged groups. To some extent these trends may reflect changing attitudes among health professionals. However, the increasing excess perinatal mortality observed both in the non-western and the short-educated groups, is disquieting and may indicate that these women as a group are more vulnerable than one generation ago. This is supported by the prevalence of low birth weight.

A strength of the present study is the large study population which provided statistical power. The variables were well defined and acquired from official registers with low occurrence of misclassification, which, to the extent it exists, would be non-differential. Education is a reliable indicator of SE status since it is clearly related to income, occupation, living conditions, social integration, lifestyle, quality of life, burden of disease and health in general.^2^^,^^5^^,^^29^ The long observation period made it possible to assess secular trends in the provision of health care to different groups of women.

Our results might have been influenced by a number of confounders. Obviously, the association between SE group and pathology will influence the needs of care. The stratification into term and preterm births to some extent reduced the confounding. Maternal age, parity and year of birth were adjusted for as potential confounders. The size of the maternity unit would have different effects in the short-educated and the non-western groups. Women living in remote areas generally have shorter education and deliver in smaller maternity units in which transfer to an NICU or other services are more complicated. This would cause a spurious association between short education and low provision of care. Non-western women would live in urban areas and deliver at larger maternity units well equipped with health services. In this group, a reported low provision of care would represent a conservative estimate. To avoid such confounding, we adjusted for size of maternity unit.

Whether our findings apply in other countries is unclear, but as SE gradients in the distribution of health services have been observed in Norway known to have comparatively low SE gradients in general, the situation may be more worrying in other countries.

In term births, we assume more pathological conditions in the short-educated women which would necessitate more frequent obstetric services. In the 1970s, this group was not provided with excess services, unlike today when this is the case. In most preterm births, need of special obstetric services is involved and more or less independent of social background. In these births, the short-educated women were initially also provided with less frequent services, as opposite to what was observed in more recent years when equal services were provided across educational groups. It is interesting to note that this secular trend in the distribution of obstetric health care did not result in a reduction of care in the women with longer education.

In a number of studies, a higher perinatal mortality has been documented among children of women with short education.^13^^,^^30^ A similar gradient was observed in the present study. To the extent that mortality is dependent on medical care, one would expect that both the improvement and the redistribution of obstetric care observed in Norway during the observation period would reduce the gradient in perinatal mortality. This was not the case. In preterm births, excess perinatal mortality increased both in the short-educated and the non-western groups. In term births, it remained high in the short-educated group and increased in the non-western group. In the interpretation of these results, we have to consider that the short education group has become much smaller and more marginalized during the study period. Consequently, the group will be more exposed to risk factors and thereby suffer an increasing perinatal mortality.

The situation is different for non-western women. They represent an increasing group of births. The group is heterogeneous, comprising different cultural, religious and social backgrounds, as well as individual factors related to life style and living conditions. This makes it difficult to identify specific risk factors causing the excess perinatal mortality. Still, it seems reasonable to suggest that the needs for more care in these births are not met by the beneficial redistribution of the obstetric services observed in Norway.

Technological development represents a factor contributing to the change in provision of health care observed since 1970s. Obstetric pain relief is an example. Epidural analgesia was implemented in obstetric care in the 1970s. In the beginning, the use was restricted and thereby limited to a smaller group; women with long education were overrepresented. Throughout the observation period, the use of epidural analgesia increased to involve more than 20% of all births, and eventually the use was quite equally distributed between the groups. The increase in availability seemed to even out the inequalities, with less discrimination as a result.

During the observation period, the CS-rate increased from 3.8% in 1967–70 to 15.5% in 2001–09. This may be accounted for by technological development as well as an increasing maternal age, but the number of CSs performed on maternal request demand has also increased.^31^^,^^32^ In these pregnancies, medical indications for a CS are rare, which involves a possibility of an SE gradient. However, we observed a ‘higher’ CS rate in the short-educated group throughout the observation period limited to term births. A higher occurrence of pathology in short-educated groups may explain the high CS rate, consistent with a more frequent transfer to an NICU. In non-western women, the excess CS-rate decreased through the years. Many other countries have a higher CS-rate than Norway,^33^ which may account for the observed difference and trend.

In addition to occurrence of pathology and attitudes among health professionals, several other mechanisms may be involved in the processes by which health needs are met by the health services. Adequate treatment depends on the patient acknowledging a problem and seeking medical attention, communication, mutual understanding and finally the type of intervention involved.

Our results suggest a challenge to antenatal care, which needs to be addressed, most likely both by educating the parents and in the training of health professionals. Still, the fact that the RRs involved approached unity and equality towards the end of the observation period was gratifying.

## Conclusion

Our study suggests a favourable development during the last decades in the distribution of obstetric health services in Norway between different SE groups. While medical interventions in the 1970s were employed less in women of short than long education, the employment in women with short education increased to equal or even higher levels towards the end of the period. The same trend was observed for non-western women. Still, the excess perinatal mortality in both the short-educated and the non-western groups remained. The results suggest increasing perinatal health problems in vulnerable groups and indicate need for a closer obstetric follow-up and surveillance. Midwives and general practitioners should be aware of risk factors and should offer support, education and more frequent contacts. Further studies, addressing distribution of antenatal care would provide a basis for developing preventive measures aiming at SE equality.

## Ethical approval

The study was approved by the internal review board of the Medical Birth Registry of Norway and by the regional ethics committee, REK Vest, Norway (2009/1868).

*Conflicts of interest*: All authors have completed the Unified Competing Interest form at www.icmje.org/coi_disclosure.pdf (available from the corresponding author).

**Key points**

- During the study period 1967–2009 the distribution of obstetric services moved in a beneficial direction with more services provided to less privileged groups.
- Perinatal mortality remained almost twice as high among children of short-educated women and non-western immigrants throughout the study period.
- Even though the study suggests a better distribution of obstetric health services across socioeconomic groups, the need for obstetric follow-up and surveillance seems to increase among vulnerable groups.
